# Supplementary material for: Immunoproximity biotinylation reveals the axon initial segment proteome
Source: Nat Commun. 2023 Dec 11;14:8201. doi: 10.1038/s41467-023-44015-2 (PMC10713531; doi:10.1038/s41467-023-44015-2)
Supplement: Supplementary file 1 — Supplementary information [file 41467_2023_44015_MOESM1_ESM.pdf]

**SUPPLEMENTARY INFORMATION**

# Immunoproximity biotinylation reveals the axon initial segment proteome

Wei Zhang *et al*.

**Supplementary information includes**: supplementary figures 1-5

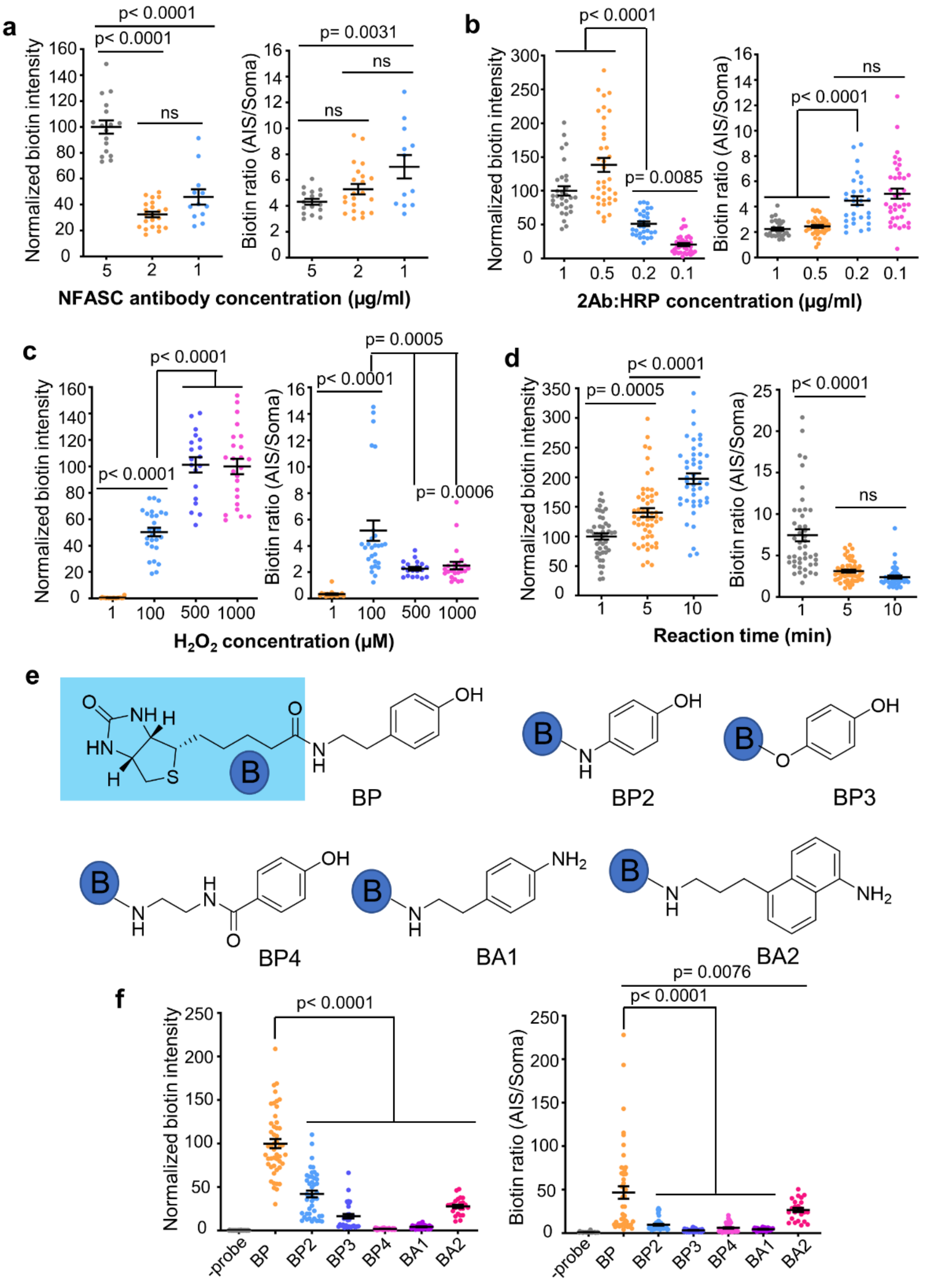


**Supplementary Figure 1. Optimization of anti-NFASC directed proximity labeling.**

**a-f**, Anti-NFASC antibody directed proximity labeling parameters were tested in DIV10-14 fixed cortical neurons. Tested parameters are as follows: first antibody (1Ab, a), HRP conjugated second antibody (2Ab, b) and $H_2O_2$ concentration (c), reaction duration (d) and reaction substrates (f). The stock concentration of 1Ab and 2Ab is 1 mg/ml. (e) Chemical probes tested in this study. The labeling efficiency was evaluated by the mean intensity of

biotinylated proteins signal in the AIS. The labeling specificity was evaluated by the ratio of biotinylated protein signal in the AIS against that in the soma. Each point represents one neuron. The number of quantified neurons is shown in the source data. Graphs are presented as mean ± SEM, one-way ANOVA, p-values are indicated on figures and ns, not significant.

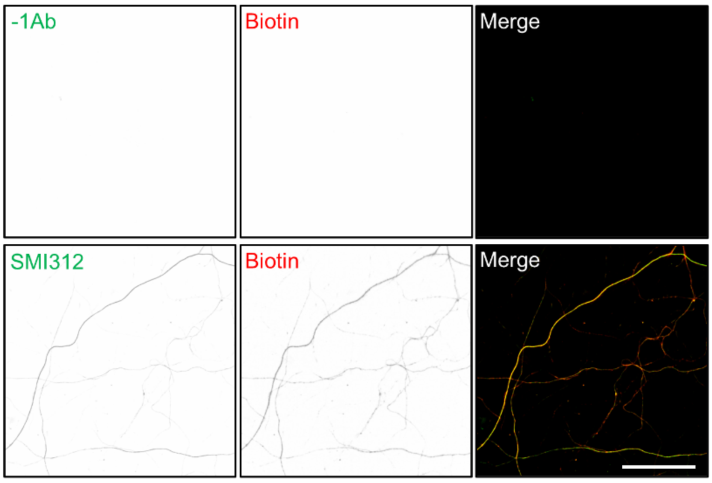


**Supplementary Figure 2. SMI312 directed proximity labeling.** Fixed DIV14 cortical neurons were processed for proximity labeling with or without SMI312 antibody. Two independent experiments were performed. Biotinylated proteins are in red, endogenous proteins labelled by SMI312 is in green. Scale bar = 50 μm.

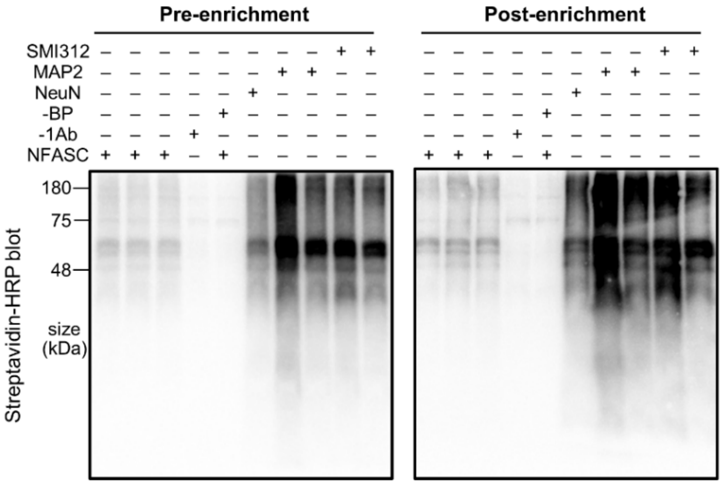


**Supplementary Figure 3. Proximity labeling and enrichment in DIV14 TMT experiments.** Streptavidin blots of DIV14 cortical neuron lysates (pre-enrichment) or streptavidin bead eluate (post-enrichment) from samples of anti-NFASC, NeuN, MAP2, SMI312 and no primary antibody (-1Ab) or substrate BP (-BP). Samples of anti-NFASC, -1Ab, -BP, anti-NeuN, anti-MAP2 and anti-SMI312 were loaded as the ratio of 3: 3: 3: 1: 1: 1, respectively. The experiment was performed once.

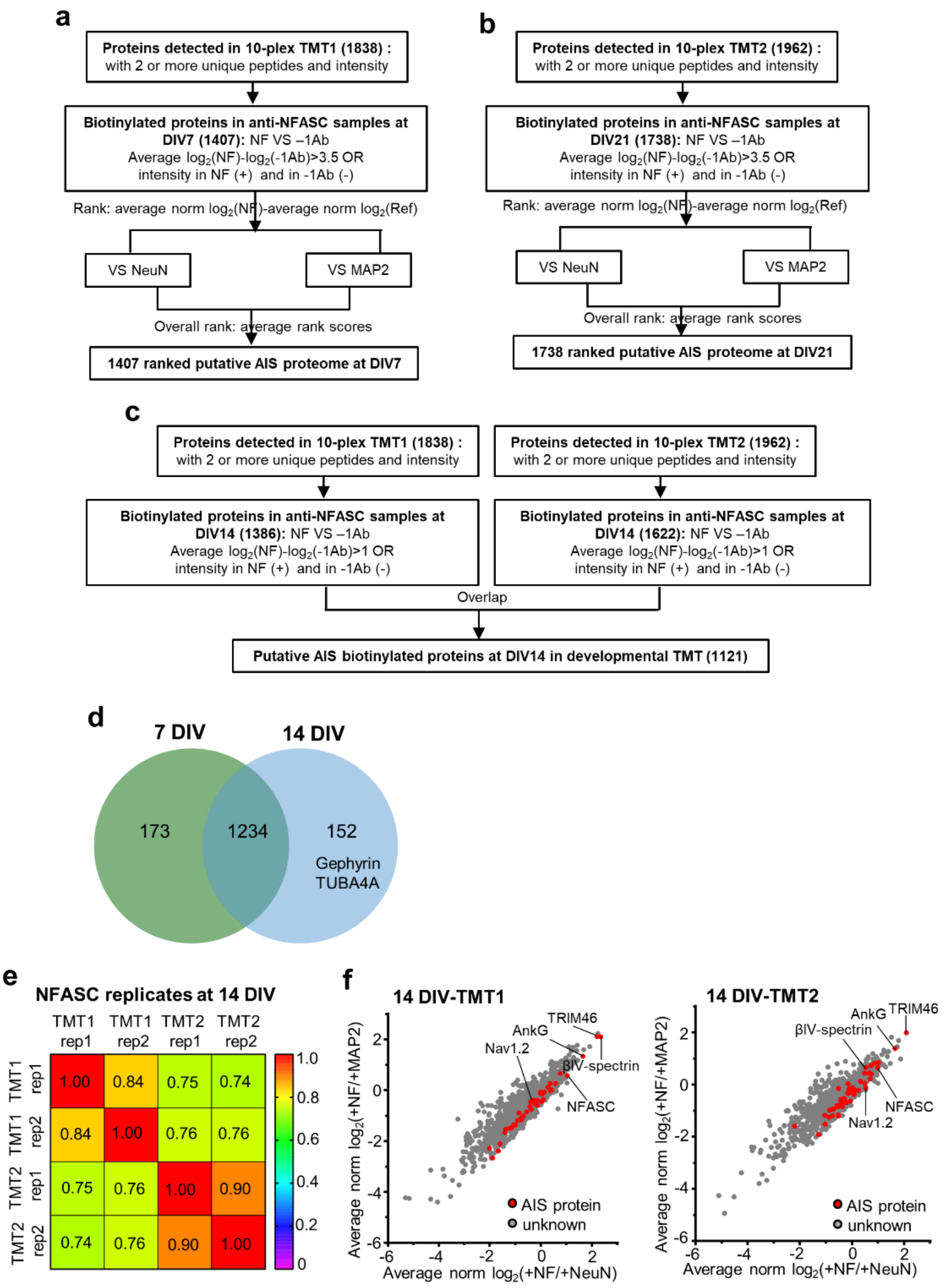


**Supplementary Figure 4. The AIS developmental proteome.**

**a-c**, Workflow for DIV7 (a), 21 (b) and 14 (c) biotinylated protein analysis in anti-NFASC samples.

**d**, Venn diagram of anti-NFASC targeted biotinylated proteins between DIV7 and 14 in 10-plex TMT1.

**e-f**, Analysis of DIV14 anti-NFASC targeted biotinylated proteins from two parallel TMT experiments. (e) Correlation analysis of biological replicates showing high reproducibility

in anti-NFASC directed AIS proximity labeling from two 10-plex TMT experiments. (f) Scatterplot showing the enrichment of anti-NFASC targeted biotinylated proteins against soma (x-axis) and somatodendrites (y-axis). Red dots indicate reported AIS components and gray dots are proteins having no AIS information.

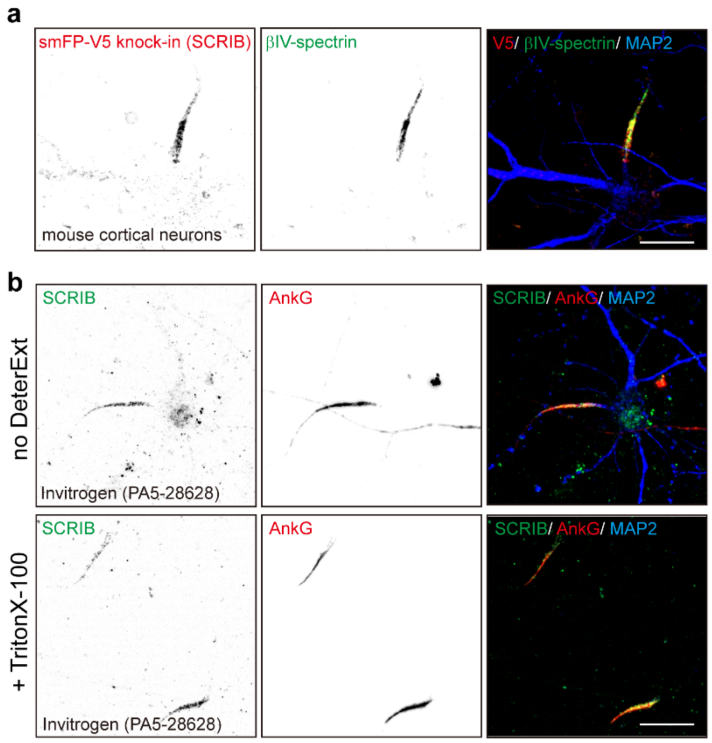


**Supplementary Figure 5. SCRIB localizes at the AIS.**

**a**, Representative image of smFP-V5-tagged SCRIB in Cas9 transgenic mouse neurons. Cortical neurons were infected at DIV0 with gRNA and donor AAV and fixed at DIV10. Two independent experiments were performed. Samples were stained for the V5 tag (red, endogenous SCRIB), βIV-spectrin (green, AIS), and MAP2 (blue, somatodendrites). Scale bar = 20 μm.

**b**, Representative images of DIV14 rat neurons stained with the Invitrogen SCRIB antibody targeting amino acids 1568-1630. Two independent experiments were performed. Hippocampal neurons were treated with or without 0.5% TritonX-100 before fixation and stained for SCRIB (green), AnkG (red, AIS), and MAP2 (blue, somatodendrites). Scale bar = 20 μm.
