# Supplementary material for: Immunoproximity biotinylation reveals the axon initial segment proteome
Source: Nat Commun. 2023 Dec 11;14:8201. doi: 10.1038/s41467-023-44015-2 (PMC10713531; doi:10.1038/s41467-023-44015-2)
Supplement: Supplementary file 3 — Description of Additional Supplementary Files [file 41467_2023_44015_MOESM3_ESM.pdf]

Description of Additional Supplementary Data

File name: Supplementary Data 1
Description: AIS proteome pilot experiments with dimethyl labeling

File name: Supplementary Data 2
Description: AIS proteome at DIV14 with 10-plex TMT labeling

File name: Supplementary Data 3
Description: AIS proteome across neuronal maturation
