# Supplementary material for: Immunoproximity biotinylation reveals the axon initial segment proteome
Source: Nat Commun. 2023 Dec 11;14:8201. doi: 10.1038/s41467-023-44015-2 (PMC10713531; doi:10.1038/s41467-023-44015-2)
Supplement: Supplementary file 7 — Reporting Summary [file 41467_2023_44015_MOESM7_ESM.pdf]

nature portfolio

Corresponding author(s): Rasband

Last updated by author(s): Nov 16, 2023

Reporting Summary

Nature Portfolio wishes to improve the reproducibility of the work that we publish. This form provides structure for consistency and transparency in reporting. For further information on Nature Portfolio policies, see our Editorial Policies and the Editorial Policy Checklist.

Statistics

For all statistical analyses, confirm that the following items are present in the figure legend, table legend, main text, or Methods section.

n/a	Confirmed	
☐	☒	The exact sample size (*n*) for each experimental group/condition, given as a discrete number and unit of measurement
☐	☒	A statement on whether measurements were taken from distinct samples or whether the same sample was measured repeatedly
☐	☒	The statistical test(s) used AND whether they are one- or two-sided<br>*Only common tests should be described solely by name; describe more complex techniques in the Methods section.*
☒	☐	A description of all covariates tested
☒	☐	A description of any assumptions or corrections, such as tests of normality and adjustment for multiple comparisons
☐	☒	A full description of the statistical parameters including central tendency (e.g. means) or other basic estimates (e.g. regression coefficient) AND variation (e.g. standard deviation) or associated estimates of uncertainty (e.g. confidence intervals)
☐	☒	For null hypothesis testing, the test statistic (e.g. F, t, r) with confidence intervals, effect sizes, degrees of freedom and P value noted<br>*Give P values as exact values whenever suitable.*
☒	☐	For Bayesian analysis, information on the choice of priors and Markov chain Monte Carlo settings
☒	☐	For hierarchical and complex designs, identification of the appropriate level for tests and full reporting of outcomes
☐	☒	Estimates of effect sizes (e.g. Cohen's d, Pearson's r), indicating how they were calculated

Our web collection on statistics for biologists contains articles on many of the points above.

Software and code

Policy information about availability of computer code

Data collection	1. The Orbitrap Fusion LUMOS Tribrid Mass Spectrometer was used for LC-MS/MS data collection.<br>2. The ChemiDoc MP Imaging System (Bio-Rad) was used for immunoblotting and silver staining imaging.<br>3. For imaging, the following tools and software were used: Inverted fluorescence microscope (Nikon-TiE, Japan) equipped with scientific CMOS cameras (Hamamatsu ORCA-Flash 4.0 v2), LabVIEW (National Instruments), Zeiss AxioImager Z2 fitted with apotome microscope, Zeiss ZEN software (Blue Edition), STED microscope using a STEDyCON system fitted to a Nikon Eclipse Ti2 microscope, and Huygens Essential (version 21.10).
Data analysis	1. The MaxQuant software (version 1.6.10.43) was used for LC-MS/MS raw data analysis.<br>2. The Fiji-ImageJ (NIH) software was used for intensity and periodicity analysis.<br>3. The GraphPad Prism 8 software was used for statistical analysis.<br>4. R studio (version 4.2.0, The R Foundation, https://www.rstudio.com/) was used for heatmap analysis.

For manuscripts utilizing custom algorithms or software that are central to the research but not yet described in published literature, software must be made available to editors and reviewers. We strongly encourage code deposition in a community repository (e.g. GitHub). See the Nature Portfolio guidelines for submitting code & software for further information.

Data

Policy information about availability of data

All manuscripts must include a data availability statement. This statement should provide the following information, where applicable:

- Accession codes, unique identifiers, or web links for publicly available datasets
- A description of any restrictions on data availability
- For clinical datasets or third party data, please ensure that the statement adheres to our policy

1. All data generated or analyzed during this study are included in this published article. Raw data of proteome files have been deposited in the ProteomeXchange database under the accession code PXD045921. Original proteomics datasets from MaxQuant and analyzed source data underlying related figures in this study are included as Supplementary Data 1-3 and a source data file.
2. Gene Ontology cellular compartment analysis utilized annotation database of the Gene Ontology Resource (https://geneontology.org/).

Research involving human participants, their data, or biological material

Policy information about studies with human participants or human data. See also policy information about sex, gender (identity/presentation), and sexual orientation and race, ethnicity and racism.

Reporting on sex and gender	N/A
Reporting on race, ethnicity, or other socially relevant groupings	N/A
Population characteristics	N/A
Recruitment	N/A
Ethics oversight	N/A

Note that full information on the approval of the study protocol must also be provided in the manuscript.

Field-specific reporting

Please select the one below that is the best fit for your research. If you are not sure, read the appropriate sections before making your selection.

☒ Life sciences ☐ Behavioural & social sciences ☐ Ecological, evolutionary & environmental sciences

For a reference copy of the document with all sections, see nature.com/documents/nr-reporting-summary-flat.pdf

Life sciences study design

All studies must disclose on these points even when the disclosure is negative.

Sample size

Sample size n were independent experiments or animals. In some experiments, n was the number of cells or AIS counted in a given experiment. No statistical tests were performed to predetermine the sample size as the research is the original and exploratory study.

Data exclusions

In the case of dimethyl experiments, contaminants and proteins identified as reversed hits were removed. Proteins containing at least 2 unique peptides and quantified in at least two out of three independent replicates were retained for further analysis (anti-NFASC vs. anti-NeuN: 965; anti-NFASC vs. anti-MAP2: 802). Additionally, an H/L ratio > 1.5 was set as the cutoff for each replicate in the experiment of anti-NFASC versus no primary antibody, which resulted in the identification of 704 proteins. Proteins in three pairs were intersected and a total of 568 proteins were obtained.
In the case of DIV14 TMT experiments, contaminants and proteins identified as reversed hits were removed. Proteins with at least two unique peptides were kept. Ratios were calculated by division using their MS intensity. The average ratio of replicates was used for the following analysis. The cutoff ratio was set at 2 for +NFASC/-BP and +NFASC/-1Ab. A total of 1403 proteins were retained for further analysis.
In the case of AIS developmental TMT experiments, two parallel 10-plex TMT labeling were employed to analyze AIS developing (DIV7), mature (DIV14) and completely mature (DIV21) proteome changes. Contaminants and proteins identified as reversed hits were removed. Proteins with at least two unique peptides were kept. Ratios were calculated by division using their MS intensity. The average ratio of replicates was used for further analysis. Proteins were filtered with the standard of 'average log2(+NFASC)-log2(-NFASC)>3.5' for DIV7 and 21, while for DIV14 the standard was set as 'average log2(+NFASC)-log2(-NFASC)>1'. This setting was based on the endogenous biotinylated proteins distribution. Proteins with intensity in +NFASC condition, not in -NFASC condition were also retained.
In the case of other data quantifications, no data were excluded from the analyses.

Replication

Both biological and technical replicates were performed. The number of independent experiments/ samples for replication is described in the methods or figure legends. The reproducibility of our proteomic data was confirmed by parallel analysis using Pearson correlation. Three independent experiments were performed for dimethyl proteome in our pilot experiment. To improve proteome data quality and

	quantification, samples from different dishes and independent experiments were collected and arranged in the same 10-plex TMT LC-MS/MS. At least two replicates were prepared for positive conditions.
Randomization	Samples were randomly allocated and processed following the requirements in different conditions.
Blinding	The investigators were not blinded to group allocation in this study.

Behavioural & social sciences study design

All studies must disclose on these points even when the disclosure is negative.

Study description	*Briefly describe the study type including whether data are quantitative, qualitative, or mixed-methods (e.g. qualitative cross-sectional, quantitative experimental, mixed-methods case study).*
Research sample	*State the research sample (e.g. Harvard university undergraduates, villagers in rural India) and provide relevant demographic information (e.g. age, sex) and indicate whether the sample is representative. Provide a rationale for the study sample chosen. For studies involving existing datasets, please describe the dataset and source.*
Sampling strategy	*Describe the sampling procedure (e.g. random, snowball, stratified, convenience). Describe the statistical methods that were used to predetermine sample size OR if no sample-size calculation was performed, describe how sample sizes were chosen and provide a rationale for why these sample sizes are sufficient. For qualitative data, please indicate whether data saturation was considered, and what criteria were used to decide that no further sampling was needed.*
Data collection	*Provide details about the data collection procedure, including the instruments or devices used to record the data (e.g. pen and paper, computer, eye tracker, video or audio equipment) whether anyone was present besides the participant(s) and the researcher, and whether the researcher was blind to experimental condition and/or the study hypothesis during data collection.*
Timing	*Indicate the start and stop dates of data collection. If there is a gap between collection periods, state the dates for each sample cohort.*
Data exclusions	*If no data were excluded from the analyses, state so OR if data were excluded, provide the exact number of exclusions and the rationale behind them, indicating whether exclusion criteria were pre-established.*
Non-participation	*State how many participants dropped out/declined participation and the reason(s) given OR provide response rate OR state that no participants dropped out/declined participation.*
Randomization	*If participants were not allocated into experimental groups, state so OR describe how participants were allocated to groups, and if allocation was not random, describe how covariates were controlled.*

Ecological, evolutionary & environmental sciences study design

All studies must disclose on these points even when the disclosure is negative.

Study description	*Briefly describe the study. For quantitative data include treatment factors and interactions, design structure (e.g. factorial, nested, hierarchical), nature and number of experimental units and replicates.*
Research sample	*Describe the research sample (e.g. a group of tagged Passer domesticus, all Stenocereus thurberi within Organ Pipe Cactus National Monument), and provide a rationale for the sample choice. When relevant, describe the organism taxa, source, sex, age range and any manipulations. State what population the sample is meant to represent when applicable. For studies involving existing datasets, describe the data and its source.*
Sampling strategy	*Note the sampling procedure. Describe the statistical methods that were used to predetermine sample size OR if no sample-size calculation was performed, describe how sample sizes were chosen and provide a rationale for why these sample sizes are sufficient.*
Data collection	*Describe the data collection procedure, including who recorded the data and how.*
Timing and spatial scale	*Indicate the start and stop dates of data collection, noting the frequency and periodicity of sampling and providing a rationale for these choices. If there is a gap between collection periods, state the dates for each sample cohort. Specify the spatial scale from which the data are taken*
Data exclusions	*If no data were excluded from the analyses, state so OR if data were excluded, describe the exclusions and the rationale behind them, indicating whether exclusion criteria were pre-established.*
Reproducibility	*Describe the measures taken to verify the reproducibility of experimental findings. For each experiment, note whether any attempts to repeat the experiment failed OR state that all attempts to repeat the experiment were successful.*
Randomization	*Describe how samples/organisms/participants were allocated into groups. If allocation was not random, describe how covariates were controlled. If this is not relevant to your study, explain why.*

Blinding

Describe the extent of blinding used during data acquisition and analysis. If blinding was not possible, describe why OR explain why blinding was not relevant to your study.

Did the study involve field work? ☐ Yes ☒ No

Reporting for specific materials, systems and methods

We require information from authors about some types of materials, experimental systems and methods used in many studies. Here, indicate whether each material, system or method listed is relevant to your study. If you are not sure if a list item applies to your research, read the appropriate section before selecting a response.

Materials & experimental systems

n/a	Involved in the study
☐	☒ Antibodies
☐	☒ Eukaryotic cell lines
☒	☐ Palaeontology and archaeology
☐	☒ Animals and other organisms
☒	☐ Clinical data
☒	☐ Dual use research of concern
☒	☐ Plants

Methods

n/a	Involved in the study
☒	☐ ChIP-seq
☒	☐ Flow cytometry
☒	☐ MRI-based neuroimaging

Antibodies

Antibodies used

Antibodies used for immunocytochemistry were: mouse anti-Pan-Neurofascin (NeuroMab, Cat# 75-172, 1:1000), mouse anti-AnkG (NeuroMab, Cat# 75-146, 1:1000), chicken anti-AnkG (Synaptic Systems, Cat# 386006, 1:500), guinea pig anti-AnkG (Synaptic Systems, Cat# 386004, 1:1000), mouse anti-NeuN (Abcam, Cat# ab104224, 1:1000), mouse anti-Neurofilament (Biolegend, Cat# 837904, 1:1000), mouse anti-MAP2 (Sigma-Aldrich, Cat# M1406, 1:1000), chicken anti-MAP2 (Abcam, Cat# ab5392, 1:10000), chicken anti-MAP2 (EnCor Biotechnology, Cat# CPCA-MAP2, 1: 2000), chicken anti-TRIM46 (Synaptic Systems, Cat# 377006, 1:500), rabbit anti-βIV-spectrin (laboratory of Dr. Matthew Rasband in BCM, 1:1000), rabbit anti-SCRIB (Abclonal, Cat# A17450, 1:50), rabbit anti-SCRIB (Invitrogen, Cat# PA5-28628, 1:100), mouse anti-V5 (Invitrogen, Cat# R960CUS, 1:500), rat anti-HA (Millipore Sigma, Cat# 11867423001, 1:1000), goat anti-mouse IgG2a, Alexa Fluor 488 (Invitrogen, Cat# A21131, 1:1000), goat anti-mouse IgG (H+L), Alexa Flour 488 (Invitrogen, Cat# A11029, 1:1000), 568 (Invitrogen, Cat# A11031, 1:1000), and Plus 594 (Invitrogen, Cat# A32742, 1:1000), goat anti-rabbit IgG (H+L), Alexa Flour 488 (Invitrogen, Cat# A11034, 1:1000), and 568 (Invitrogen, Cat# A11036, 1:1000), goat anti-rat IgG (H+L), Alexa Flour 594 (Invitrogen, Cat# A11007, 1:1000), goat anti-chicken IgY (H+L), Alexa Flour 488 (Invitrogen, Cat# A11039, 1:1000), 647 (Invitrogen, Cat# A21449, 1:1000), and AMCA (Jackson, Cat# 103-155-155, 1:200), Streptavidin conjugated with Alexa Fluor 568 (Invitrogen, Cat# S11226, 1:1000) or 647 (Invitrogen, Cat# S21374, 1:1000).
Antibodies used for immunohistochemistry were: mouse anti-AnkG (NeuroMab, Cat# 75-146, 1:500), rabbit anti-SCRIB (Abclonal, Cat# A17450, 1:50), mouse anti-V5 (Invitrogen, Cat# R960CUS, 1:500), and rabbit anti-βIV-spectrin (laboratory of Dr. Matthew Rasband in BCM, 1:500), goat anti-mouse IgG (H+L), Alexa Flour 568 (Invitrogen, Cat# A11031, 1:1000), goat anti-rabbit IgG (H+L), Alexa Flour 488 (Invitrogen, Cat# A11034, 1:1000), and 568 (Invitrogen, Cat# A11036, 1:1000), and goat anti-mouse IgG2a, Alexa Fluor 488 (Invitrogen, Cat# A21131, 1:1000).
Antibodies used for immunoblotting were: rabbit anti-GFP (Invitrogen, Cat# A11122, RRID: AB_221569, 1:1000), chicken anti-GFP (Aves Labs, Cat# GFP-1020, RRID: AB_2307313, 1:2000), mouse anti-Flag (MBL, Cat# M185-3L, 1:1000), chicken anti-mCherry (Aves Labs, Cat# MCHERRY-0020, 1:1000), rabbit anti-Flag conjugated with HRP (Cell Signaling, Cat# 86861, 1:1000), streptavidin-HRP (Invitrogen, Cat# 21124, 1:5000), goat anti-mouse IgG (H+L) conjugated with HRP (Jackson, Cat# 115-035-146, 1:5000), goat anti-rabbit IgG (H+L) conjugated with HRP (Jackson, Cat# 111-035-003, 1:5000), and goat anti-chicken IgY conjugated with HRP (Aves, Cat# H-1004, 1:5000).

Validation

rabbit anti-βIV-spectrin antibody (laboratory of Dr. Matthew Rasband in BCM) is knockout validated by immunoblotting and immunohistology. See Yang Y et al., J Neurosci. 2004;24(33):7230-7240.
guinea pig anti-AnkG antibody (Synaptic Systems, Cat# 386004) is validated by CRISPR-Cas9 mediated gRNA knockout in cultured rat neurons in this paper.
rabbit anti-SCRIB antibody (Abclonal, Cat# A17450) is validated by CRISPR-Cas9 mediated gRNA knockout in cultured rat neurons in this paper.
rabbit anti-SCRIB antibody (Invitrogen, Cat# PA5-28628) is advanced verified by knockdown, immunoblotting and immunofluorescence (https://www.thermofisher.com/antibody/product/SCRIB-Antibody-Polyclonal/PA5-28628). In cultured rat neurons similar results were obtained as Abclonal SCRIB antibody labeling in this paper.
Following 9 antibodies were validated by immunolabeling and/ or immunoblotting consistent with providers' data sheets. chicken anti-AnkG (Synaptic Systems, Cat# 386006): https://www.sysy.com/product/386006#list; mouse anti-NeuN (Abcam, Cat# ab104224): https://www.abcam.com/products/primary-antibodies/neun-antibody-1b7-neuronal-marker-ab104224.html; mouse anti-Neurofilament (Biolegend, Cat# 837904): https://www.biolegend.com/en-us/explore-new-products/purified-anti-neurofilament-marker-pan-axonal-cocktail-12811?GroupID=BLG15643; mouse anti-MAP2 (Sigma-Aldrich, Cat# M1406): https://www.sigmaaldrich.com/US/en/product/sigma/m1406; chicken anti-MAP2 (Abcam, Cat# ab5392): https://www.abcam.com/products/primary-antibodies/map2-antibody-ab5392.html; chicken anti-MAP2 (EnCor Biotechnology, Cat# CPCA-MAP2): https://encorbio.com/product/cpca-map2ab/; chicken anti-TRIM46 (Synaptic Systems, Cat# 377006): https://www.sysy.com/product/377006#list; mouse anti-Pan-Neurofascin (NeuroMab, Cat# 75-172) and mouse anti-AnkG (NeuroMab, Cat# 75-146):

https://neuromab.ucdavis.edu/.
Antibodies mouse anti-V5 (Invitrogen, Cat# R960CUS), rat anti-HA (Millipore Sigma, Cat# 11867423001), rabbit anti-GFP (Invitrogen, Cat# A11122), chicken anti-GFP (Aves Labs, Cat# GFP-1020), mouse anti-Flag (MBL, Cat# M185-3L), chicken anti-mCherry (Aves Labs, Cat# MCHERRY-0020), and rabbit anti-Flag conjugated with HRP (Cell Signaling, Cat# 86861) were validated by plasmids overexpression fused with these tags, followed by immunofluorescence and immunoblotting.
All second antibodies were validated by binding test against their target primary antibody species without cross-reactivity against other species.

Eukaryotic cell lines

Policy information about cell lines and Sex and Gender in Research

Cell line source(s)	HEK293T cells were a gift from Dr. Benjamin Arenkiel in Baylor College of Medicine.
Authentication	Cell lines were not authenticated.
Mycoplasma contamination	Cell lines were not tested for mycoplasma contamination.
Commonly misidentified lines (See ICLAC register)	No commonly misidentified cell lines were used in this study.

Palaeontology and Archaeology

Specimen provenance	*Provide provenance information for specimens and describe permits that were obtained for the work (including the name of the issuing authority, the date of issue, and any identifying information). Permits should encompass collection and, where applicable, export.*
Specimen deposition	*Indicate where the specimens have been deposited to permit free access by other researchers.*
Dating methods	*If new dates are provided, describe how they were obtained (e.g. collection, storage, sample pretreatment and measurement), where they were obtained (i.e. lab name), the calibration program and the protocol for quality assurance OR state that no new dates are provided.*

☐ Tick this box to confirm that the raw and calibrated dates are available in the paper or in Supplementary Information.

Ethics oversight	*Identify the organization(s) that approved or provided guidance on the study protocol, OR state that no ethical approval or guidance was required and explain why not.*

Note that full information on the approval of the study protocol must also be provided in the manuscript.

Animals and other research organisms

Policy information about studies involving animals; ARRIVE guidelines recommended for reporting animal research, and Sex and Gender in Research

Laboratory animals	Animals used were: P0 Sprague-Dawley rat pups, E18.5 timed-pregnant Sprague-Dawley rats, postnatal day 7 C57BL/6 mice, P0 Cas9 transgenic pups.<br>Mice were maintained in a facility with 22oC temperature, 40-60% humidity and a normal 12h light/dark schedule. Animals were free to have food and water.
Wild animals	No wild animals were used.
Reporting on sex	Our findings apply to both genders.
Field-collected samples	No field-collected samples were used in this study.
Ethics oversight	All animal studies were approved by Peking University and Baylor College of Medicine Institutional Animal Care and Use Committee (IACUC). Performance was in accordance with the NIH guide for humane care and use of animals.

Note that full information on the approval of the study protocol must also be provided in the manuscript.

Clinical data

Policy information about clinical studies
All manuscripts should comply with the ICMJE guidelines for publication of clinical research and a completed CONSORT checklist must be included with all submissions.

Clinical trial registration	*Provide the trial registration number from ClinicalTrials.gov or an equivalent agency.*

Study protocol	*Note where the full trial protocol can be accessed OR if not available, explain why.*
Data collection	*Describe the settings and locales of data collection, noting the time periods of recruitment and data collection.*
Outcomes	*Describe how you pre-defined primary and secondary outcome measures and how you assessed these measures.*

Dual use research of concern

Policy information about [dual use research of concern]

Hazards

Could the accidental, deliberate or reckless misuse of agents or technologies generated in the work, or the application of information presented in the manuscript, pose a threat to:

No	Yes	
☒	☐	Public health
☒	☐	National security
☒	☐	Crops and/or livestock
☒	☐	Ecosystems
☒	☐	Any other significant area

Experiments of concern

Does the work involve any of these experiments of concern:

No	Yes	
☒	☐	Demonstrate how to render a vaccine ineffective
☒	☐	Confer resistance to therapeutically useful antibiotics or antiviral agents
☒	☐	Enhance the virulence of a pathogen or render a nonpathogen virulent
☒	☐	Increase transmissibility of a pathogen
☒	☐	Alter the host range of a pathogen
☒	☐	Enable evasion of diagnostic/detection modalities
☒	☐	Enable the weaponization of a biological agent or toxin
☒	☐	Any other potentially harmful combination of experiments and agents

Plants

Seed stocks	N/A
Novel plant genotypes	N/A
Authentication	N/A

ChIP-seq

Data deposition

☐ Confirm that both raw and final processed data have been deposited in a public database such as GEO.

☐ Confirm that you have deposited or provided access to graph files (e.g. BED files) for the called peaks.

Data access links<br>*May remain private before publication.*	*For "Initial submission" or "Revised version" documents, provide reviewer access links. For your "Final submission" document, provide a link to the deposited data.*
Files in database submission	*Provide a list of all files available in the database submission.*

Genome browser session (e.g. UCSC)

Provide a link to an anonymized genome browser session for "Initial submission" and "Revised version" documents only, to enable peer review. Write "no longer applicable" for "Final submission" documents.

Methodology

Replicates

Describe the experimental replicates, specifying number, type and replicate agreement.

Sequencing depth

Describe the sequencing depth for each experiment, providing the total number of reads, uniquely mapped reads, length of reads and whether they were paired- or single-end.

Antibodies

Describe the antibodies used for the ChIP-seq experiments; as applicable, provide supplier name, catalog number, clone name, and lot number.

Peak calling parameters

Specify the command line program and parameters used for read mapping and peak calling, including the ChIP, control and index files used.

Data quality

Describe the methods used to ensure data quality in full detail, including how many peaks are at FDR 5% and above 5-fold enrichment.

Software

Describe the software used to collect and analyze the ChIP-seq data. For custom code that has been deposited into a community repository, provide accession details.

Flow Cytometry

Plots

Confirm that:

- [ ] The axis labels state the marker and fluorochrome used (e.g. CD4-FITC).
- [ ] The axis scales are clearly visible. Include numbers along axes only for bottom left plot of group (a 'group' is an analysis of identical markers).
- [ ] All plots are contour plots with outliers or pseudocolor plots.
- [ ] A numerical value for number of cells or percentage (with statistics) is provided.

Methodology

Sample preparation

Describe the sample preparation, detailing the biological source of the cells and any tissue processing steps used.

Instrument

Identify the instrument used for data collection, specifying make and model number.

Software

Describe the software used to collect and analyze the flow cytometry data. For custom code that has been deposited into a community repository, provide accession details.

Cell population abundance

Describe the abundance of the relevant cell populations within post-sort fractions, providing details on the purity of the samples and how it was determined.

Gating strategy

Describe the gating strategy used for all relevant experiments, specifying the preliminary FSC/SSC gates of the starting cell population, indicating where boundaries between "positive" and "negative" staining cell populations are defined.

- [ ] Tick this box to confirm that a figure exemplifying the gating strategy is provided in the Supplementary Information.

Magnetic resonance imaging

Experimental design

Design type

Indicate task or resting state; event-related or block design.

Design specifications

Specify the number of blocks, trials or experimental units per session and/or subject, and specify the length of each trial or block (if trials are blocked) and interval between trials.

Behavioral performance measures

State number and/or type of variables recorded (e.g. correct button press, response time) and what statistics were used to establish that the subjects were performing the task as expected (e.g. mean, range, and/or standard deviation across subjects).

Acquisition

Imaging type(s) | *Specify: functional, structural, diffusion, perfusion.*

Field strength | *Specify in Tesla*

Sequence & imaging parameters | *Specify the pulse sequence type (gradient echo, spin echo, etc.), imaging type (EPI, spiral, etc.), field of view, matrix size, slice thickness, orientation and TE/TR/flip angle.*

Area of acquisition | *State whether a whole brain scan was used OR define the area of acquisition, describing how the region was determined.*

Diffusion MRI ☐ Used ☐ Not used

Preprocessing

Preprocessing software | *Provide detail on software version and revision number and on specific parameters (model/functions, brain extraction, segmentation, smoothing kernel size, etc.).*

Normalization | *If data were normalized/standardized, describe the approach(es): specify linear or non-linear and define image types used for transformation OR indicate that data were not normalized and explain rationale for lack of normalization.*

Normalization template | *Describe the template used for normalization/transformation, specifying subject space or group standardized space (e.g. original Talairach, MNI305, ICBM152) OR indicate that the data were not normalized.*

Noise and artifact removal | *Describe your procedure(s) for artifact and structured noise removal, specifying motion parameters, tissue signals and physiological signals (heart rate, respiration).*

Volume censoring | *Define your software and/or method and criteria for volume censoring, and state the extent of such censoring.*

Statistical modeling & inference

Model type and settings | *Specify type (mass univariate, multivariate, RSA, predictive, etc.) and describe essential details of the model at the first and second levels (e.g. fixed, random or mixed effects; drift or auto-correlation).*

Effect(s) tested | *Define precise effect in terms of the task or stimulus conditions instead of psychological concepts and indicate whether ANOVA or factorial designs were used.*

Specify type of analysis: ☐ Whole brain ☐ ROI-based ☐ Both

Statistic type for inference | *Specify voxel-wise or cluster-wise and report all relevant parameters for cluster-wise methods.*

(See Eklund et al. 2016)

Correction | *Describe the type of correction and how it is obtained for multiple comparisons (e.g. FWE, FDR, permutation or Monte Carlo).*

Models & analysis

n/a	Involved in the study
☐	☐ Functional and/or effective connectivity
☐	☐ Graph analysis
☐	☐ Multivariate modeling or predictive analysis

Functional and/or effective connectivity | *Report the measures of dependence used and the model details (e.g. Pearson correlation, partial correlation, mutual information).*

Graph analysis | *Report the dependent variable and connectivity measure, specifying weighted graph or binarized graph, subject- or group-level, and the global and/or node summaries used (e.g. clustering coefficient, efficiency, etc.).*

Multivariate modeling and predictive analysis | *Specify independent variables, features extraction and dimension reduction, model, training and evaluation metrics.*
